# Wet chemical development of CuO/GO nanocomposites: its augmented antimicrobial, antioxidant, and anticancerous activity

**DOI:** 10.1007/s10856-021-06612-9

**Published:** 2021-12-11

**Authors:** Kunal Biswas, Yugal Kishore Mohanta, Awdhesh Kumar Mishra, Abdullah G. Al-Sehemi, Mehboobali Pannipara, Avik Sett, Amra Bratovcic, Debashis De, Bibhu Prasad Panda, Satya Kumar Avula, Tapan Kumar Mohanta, Ahmed Al-Harrasi

**Affiliations:** 1 Department of Biotechnology, Maulana Abul Kalam Azad University of Technology, Kolkata, West Bengal 741249 India; 2 Department of Applied Biology, School of Biological Sciences, University of Science and Technology Meghalaya, Ri-Bhoi, 793101 India; 3 Department of Biotechnology, Yeungnam University, Gyeongsan, Gyeongsangbuk-do Republic of Korea; 4 Department of Chemistry, King Khalid University, Abha, 61413 Saudi Arabia; 5 Department of Electronics and Electrical Communication Engineering, IIT Kharagpur, Kharagpur, 721302 India; 6 Department of Physical Chemistry and Electrochemistry, Faculty of Technology, University of Tuzla, Univerzitetska 8, 75000 Tuzla, Bosnia and Herzegovina; 7 Department of Computer Science and Engineering, Maulana Abul Kalam Azad University of Technology, Kolkata, West Bengal 741249 India; 8 Centre for Environmental Sciences, Siksha O Anusandhan University, Bhubaneswar, India; 9 Natural and Medical Sciences Research Centre, University of Nizwa, Nizwa, 616 Oman

**Keywords:** CuO/GO nanocomposite, Biomedical activities, Electro-mechanical device, Nanomedicine

## Abstract

This study employed a bottom-up technique to synthesize copper oxide (CuO) nanoparticles over hydrophilic graphene oxide (GO) nanosheets. The CuO/GO nanocomposite has been prepared using two selected precursors of copper nitrate and citric acid with an intermittent mixing of GO solutions. The synthesized Nanocomposites were characterized using different biophysical techniques like FT-IR, NMR, FE-SEM, and HR-TEM analyses. FT-IR analyses confirm the nanocomposites’ successful formation, which is evident from the functional groups of C=C, C-O, and Cu-C stretching vibrations. Morphological analyses reveal the depositions of CuO nanoparticles over the planar rough GO sheets, which has been elucidated from the FE-SEM and HR-TEM analyses supported by respective EDAX analyses. The antimicrobial activities have been evident from the surface roughness and damages seen from the FE-SEM analyses. The CuO/GO sheets were tested against Gram-positive (e.g., *Staphylococcus aureus*) and Gram-negative (*Escherichia coli, Pseudomonas aeruginosa*). It is evident that the intrinsic antibacterial activity of CuO/GO sheets, when combined in equal proportions, elicited a robust antibacterial activity when tested over Gram –ve representative bacteria *Escherichia coli*. The antioxidant behaviour of synthesized CuO/GO nanocomposite was evaluated by scavenging the free radicals of DPPH and ABTS. Moreover, the cytotoxic activity was also studied against epidermoid carcinoma cell line A-431. A brief mathematical formulation has been proposed in this study to uncover the possibilities of using the nanocomposites as potential drug candidates in theranostic applications in disease treatment and diagnosis. This study would help uncover the electronic properties that play in the nano-scaled system at the material-bio interface, which would aid in designing a sensitive nano-electromechanical device bearing both the therapeutic and diagnostic attributes heralding a new horizon in the health care systems.

## Abstract (web summary)

A bottom-up technique has been employed to synthesize copper oxide (CuO) nanoparticles over hydrophilic graphene oxide (GO) nanosheets. The synthesized CuO/GO nanocomposites exhibited potential antibacterial (human pathogens), antioxidant (DPPH & ABTS) and anticancer (epidermoid carcinoma A-431) activity. This study would help uncover the electronic properties that play in the nano-scaled system at the material-bio interface, which would aid in designing a sensitive nano-electromechanical device bearing both the therapeutic and diagnostic attributes heralding a new horizon in the health care systems.

## Highlights

- The CuO/GO nanocomposite has been prepared using two selected precursors of copper nitrate and citric acid with an intermittent mixing of GO solutions.
- CuO nanoparticles decoration on GO sheets to coincide the enrichment of activity and stability from their cooperation.
- CuO/GO nanocomposite has appreciable activity towards pathogenic bacteria, free radicals, and cancer cell lines.
- The as-prepared nanocomposite is proposed as a novel nanomaterial for theranostic activities bearing both therapeutic attributes along with diagnostic nature in the biological domain and healthcare.

## Graphical abstract

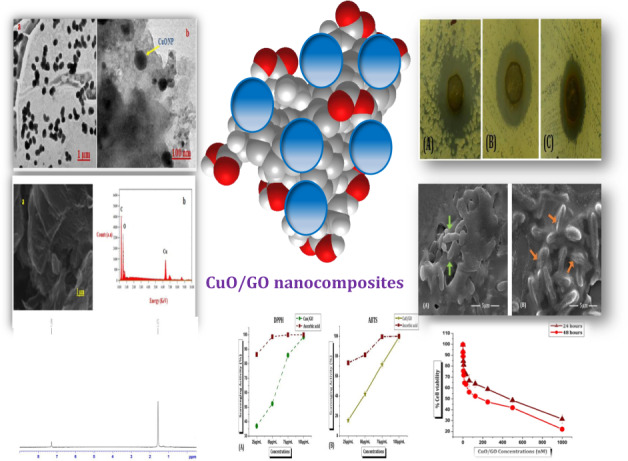

## Introduction

Owing to the intrinsic direct band gap nature, p-type attributes, robust electrochemical behaviour, and the lower cost of fabrication, the studies over Copper Oxide (CuO)nanomaterials have seen an exponential rise in different fields applications [1, 2]. Cuprous Oxide (Cu_2_O) and Cupric Oxides (CuO) are the two prevalent forms of Copper Oxide polymorphism existing in nature, forming the two most important stoichiometric compounds in the entire CuO systems [3]. The melting point of the intrinsic CuO is ~1330 °C, which is sparingly soluble in the water. The density of the pure Cupric Oxide is ~6.4 gm per cm^3^. The direct band gap value of pristine CuO nanoparticles is basically a p-type semiconductor with a value range of ~1.2–1.85 eV, attracting numerous applications from gas sensors for hydrogen, volatile organic compounds, catalysis, and especially in photovoltaic solar cells, electrochemical coatings etc. [4–8]. Owing to such widespread utility in electrochemical and sensing applications, the synthesis of CuO nanoparticles at laboratory conditions is of paramount importance. In this regard, several techniques are currently employed in the laboratory conditions for the synthesis of CuO nanoparticles like spray pyrolysis, pulsed laser deposition, plasma-based ion implantations, chemical vapour deposition etc. It has been reported that the selection of the fabrication procedures determines the underlying electrochemical, Physico-chemical, and electro-mechanical properties of the as-synthesized CuO nanoparticles [9–12]. It is well known that Graphene Oxide (GO) nanosheets are hydrophilic in nature owing to their available Oxygen groups linked with the structure, available defect attributes, which make the GO nanosheets perfectly soluble in water for different applications [13]. Several reports have been studied to ascertain the implications of GO sheets in sensor studies (Wu et al., 2021), antimicrobial studies, and several biological systems [14].

In this study, the prime focus is to extract the electrochemical, Physico-chemical attributes of the pristine CuO nanoparticles when combined and decorated in the lattice defects of the GO sheets. The nanocomposites have been studied in the context of designing a robust, novel nano and micro electro-mechanical sensing system owing to its pristine therapeutic and diagnostic properties. The study would highlight the implications of the as-synthesized CuO/GO nanocomposites into the treatment of prokaryotic systems of bacteria, which would be further validated into the in-vitro cell lines in laboratory conditions to substantiate the capabilities of the prepared nanocomposites for acting as a potential theranostic material in future.

## Material and method

### Materials

The reagents used in the study were of analytical grade and have been purchased from Sigma Aldrich and have been used without further purifications. Reagents include Copper (II) nitrate trihydrate, Sodium Hydroxide (NaOH), Potassium Chloride (KCL), Lead Nitrate and Triton-X 100 respectively. The solvent used in the entire study was double distilled water.

### Bottom up synthesis of CuO nanoparticles

In the reaction procedures, 25 ml of 0.2 mol L^−1^ Copper (II) nitrate trihydrate solution was mixed in a solution containing 25 ml of 0.3 mol L^−1^ NaOH solution, which has been thoroughly mixed into a solution containing 0.003 mol L^−1^ of Triton-X 100 with the regular and constant stirring mechanism. The resulting mixture has been autoclaved at ~200 °C for 5 h. and post vigorous stirring for ~3 h. The resulting precipitates were then washed thoroughly with the deionized water and absolute ethanol after cooling the stirred samples for 3 h. The step of precipitate washing has been followed with the effective drying in the oven at ~50 °C for 7–8 h. [15].

### Preparation of GO sheets

Graphene Oxide sheets were prepared following the conventional hummers method [16].

### Preparation of the CuO/GO nanocomposites

CuO solutions of 5 ml were mixed in a vessel containing GO solutions of 50 ml volume, which is followed by the constant stirring of the mixture solution at 100 °C for 1 h. Upon continuous stirring of the mixture solution at such temperature, it formed gel-like morphology that looks like a light, fluffy mass of both the components of CuO and GO compositions. The step is followed by annealing at 200 °C for ~2 h. The resulting product has been subjected to grinding for further characterization approaches (Bezza, F. A et al., 2020).

### Characterizations of the CuO/GO nanocomposites

The as-prepared nanocomposites of CuO/GO have been subjected to characterizations like Fourier Transform Infra-Red spectroscopy (FT-IR) (Bruker ATR) at 400 cm^−1^ to 4000 cm^−1^ scanning range for ascertaining the available functional groups in the composites. Nuclear Magnetic Resonance (NMR) has been further employed to characterize different local magnetic fields around the atomic nuclei of the nanocomposites. The spectroscopic examinations were further extended to examine morphological features of the as-prepared composite using FE-SEM and HR-TEM analyses, which is further coupled by Energy Dispersive X-Ray Spectroscopy (EDAX) analyses for the confirmation of the composite formation.

### Antibacterial Activity of the CuO/GO nanocomposites

#### Bacterial strains

The three species of human pathogenic bacteria, *Staphylococcus aureus, Escherichia coli*, and *Pseudomonas aeruginosa* were used in the antibacterial assay.

#### Micro broth dilution method and agar well diffusion method of antibacterial assays

Muller Hinton (MH) broth medium of 5 ml has been inoculated with the small colony of each test bacterial strain from its respective stock solution under appropriate aseptic conditions. The inoculated tubes were incubated overnight at 37 °C on a rotary shaker at 200 rpm. The evaluation of the antibacterial activity of the CuO/GO nanocomposites against the selected pathogenic bacteria was regulated using a well diffusion assay with Muller Hinton Agar (MHA) [17]. Briefly, 100 µl of each bacterium was grown over the processed MHA plates. Test wells of 5 mm diameter and 3 mm deep were then formed using a sterile cork borer in the inoculated agar medium. Each well was then filled with 50 µl of CuO/GO nanocomposites. The antibiotic, Gentamicin, were taken as a positive control. The respective plates were incubated at 37 °C for 24 h. The diameter of inhibition known as Zone of Inhibition (ZOI) was calculated after incubation, and a net diameter of ~ ≥8 mm was considered a positive antibacterial activity of the composites.

Antibacterial was also evaluated employing the micro broth dilution method. The minimum inhibitory concentration (MIC) of the CuO/GO nanocomposites on bacterial strains was also assessed. Additional MIC calculations were performed besides conventional MIC assays to ascertain the confirmed antibacterial activities of the nanocomposites, where inhibition proportions of ≥90% were taken into considerations that composite is eliciting augmented and remarkable antibacterial activities in the broth conditions. The test inoculum (190 µl; A_600_ = 0.1) were incubated in 10 µl of different concentrations (two-fold dilution) of the CuO/GO nanocomposites, until the level of inhibition was found to be <50%. Microplate Reader (Bio-Rad, USA) has been employed to determine the microbial growth in the conventional 96-well plates at 600 nm wavelength. Conventional Laboratory excel calculations were performed to ascertain the numerical MIC values using IC50/IC90 calculations and have been expressed as IC_50_ values. The zone of inhibition (ZOI) has been expressed in mean ± SD, and the assays were all repeated in triplicate.

#### Microscopic study of antibacterial activity

The antibacterial activity of CuO/GO nanocomposites in terms of physical interaction with pathogenic strains was studied using a Field-emission scanning electron microscope (FE-SEM).

### Antioxidant activity of CuO/GO nanocomposites

The antioxidant activity of the CuO/GO nanocomposites was evaluated by its radicle scavenging potential.

#### DPPH Radical-Scavenging Activity

The radical scavenging activity of CuO/GO nanocomposites was determined using the 1, 1-diphenyl-2-picryl-hydrazil (DPPH) assay with slight modification [18, 19]. Different concentrations (25, 50, 75 and 100 µg/ml) of CuO/GO nanocomposites were used in the assay. In the experimentations, the positive control has been designated to equivalent concentrations of Ascorbic Acid (AA), and (%) radical scavenging activity has been used to denote the effective result outcomes. The MIC for DPPH radical scavenging activity was also calculated and expressed as an IC_50_ valuation.

#### 2,2-Azino-bis (3-ethylbenzothiozoline-6-sulfonic acid) diammonium salt (ABTS) radical scavenging activity

Using well-accepted ABTS radical scavenging assays of a particular material, the activity of CuO/GO radical scavenging potential has also been determined using the previously described ABTS method [20], using different concentrations (25,50,75 and 100 μg/ml) of CuO/GO nanocomposites. Ascorbic acid at equivalent concentrations was used as a positive control. The MIC for ABTS radical scavenging activity was also calculated and expressed as an IC_50_.

### Cytotoxicity study of CuO/GO nanocomposite

#### Cell culture

Dulbecco’s Modified Eagle’s Medium (DMEM) has been used for the seeding of epidermoid carcinoma cell line A-431(NCCS, Pune, India) and has been supplemented with 10 % foetal bovine serum (FBS), which has been incubated at 37^0^C (5% CO_2_) for 24 h for the duration of the assays. The cells were trypsinized using 0.25% Trypsin-EDTA at a 70–80% assemblage. Cells were enumerated and then implanted in a 96-well Enzyme-linked immunosorbent assay (ELISA) plate at a density of 5 × 10^3^ cells/well to carry out a dimethyl thiazolyl-diphenyltetrazolium bromide (MTT) assay [21].

#### MTT Assay

CuO/GO nanocomposites were assessed for its cytotoxicity analyses using the conventional MTT Assay, wherein the cell lines were incubated with the nanocomposite for 24–48 h. incubation period. In the experimentation, 1 mg/ ml of MTT stock in PBS solution was prepared, and the doxorubicin solution was used as a positive standard. Each cultured disk was supplemented with a 500 µl MTT solution (50 μg/ ml MTT in culture medium) and has been left uncovered. The reduced formazan was extracted with the 500 μl of DMSO. The cells were incubated for 3 h. and absorbance was calculated at 595 nm wavelength in a microtiter plate reader (Bio-Rad, USA). Compared to untreated and control cells, the net cell viability has been determined using the % absorption of the treated cells [22].

### Mathematical modelling of proposed drug composites (CuO/GO) over biological targets and its pharmacodynamics analysis: a horizon for theranostic applications

#### Concentration-effect model-based drug combinations

The effectiveness of the drug interaction with the biological targets could be illustrated with the law of mass-action at the region of interaction as shown in below equation:

1 $${{{{{\mathrm{C}}}}}} + {{{{{\mathrm{R}}}}}} = \ \mathop{\leftrightarrows}\limits_{{{{{{{\mathrm{K}}}}}}_{{{{{\mathrm{b}}}}}}}}^{{{{{{{\mathrm{K}}}}}}_{{{{{\mathrm{F}}}}}}}}\ {{{{{\mathrm{RC}}}}}}$$

where, ‘C’, ‘R’ and ‘RC’ represent the drug, receptor, and drug-receptor complex, respectively.

Also, at the equilibrium, whenever the forward and backward rates are equal, then the equations stand to which is shown in below equation:

2 $$\left[ {{{{{{\mathbf{RC}}}}}}} \right]/\left[ {{{{{\mathbf{R}}}}}} \right]_{{{{{\mathbf{T}}}}}} \,= \left[ {{{{{\mathbf{C}}}}}} \right]/{{{{{\mathbf{k}}}}}}_{{{{{\mathbf{d}}}}}} + \left[ {{{{{\mathbf{C}}}}}} \right]$$

where [C] corresponds to concentration and [R]_T_ is the receptors total molar concentrations, k_d_ = k_b_/k_f_ corresponds to the drug dissociation constants and signifies the concentration at which the proposed drug binds to half of the total available receptors [18].

Also, the pharmacodynamics of the drug modelling when the drug interacts with the biological target receptors, which forms the basis of the proposed theranostic device modelling, which can detect and diagnose the available target moieties, could be illustrated by the mathematical modelling as shown in below equation:

3 $${{{{{\mathbf{E}}}}}}\left( {{{{{\mathbf{c}}}}}} \right) = {{{{{\mathbf{E}}}}}}_{{{{{{\mathbf{max}}}}}}}{{{{{\mathbf{c}}}}}}^{{{{{\mathbf{n}}}}}}/{{{{{\mathbf{EC}}}}}}^{{{{{\mathbf{n}}}}}}_{{{{{{\mathbf{50}}}}}}} + {{{{{\mathbf{c}}}}}}^{{{{{\mathbf{n}}}}}}$$

where the concentration of the drug is denoted by c, whereas the EC_5O_ denotes the concentration at which the drug exhibits half of the drug’s maximum response (E_max_). This parameter is the valuation of the drug’s potency, which is proportional to the drug’s affinity and its intrinsic efficacy. In the case of Emax, it is regulated by several drug-related aspects and associated parameters like drug efficacy, the quantitative numbers of the target receptors and the nature of relaying of the stimulus, which is required for eliciting sigmoidal drug-related responses in the desired biological targets.

In this study, the proposed nanocomposites of Copper Oxide (CuO) decorated over Graphene Oxide (GO) sheets are designed to elicit diagnostic and therapeutic activity when treated into several biological targets like cancer cell lines, bacterial targets etc.

### Statistical analysis

All the experimentations of antibacterial, cytotoxicity assays were performed in triplicate. The experimentations were determined in terms of % inhibition for antibacterial assays and % viability assays for cytotoxicity tests compared to the control sets, respectively. The experimentations were statistically determined using the Student’s *t* test of probability *p* ≤ 0.05.

## Results and Discussion

### Fourier transform infra-red spectroscopy (FT-IR)

**Fig. 1:**
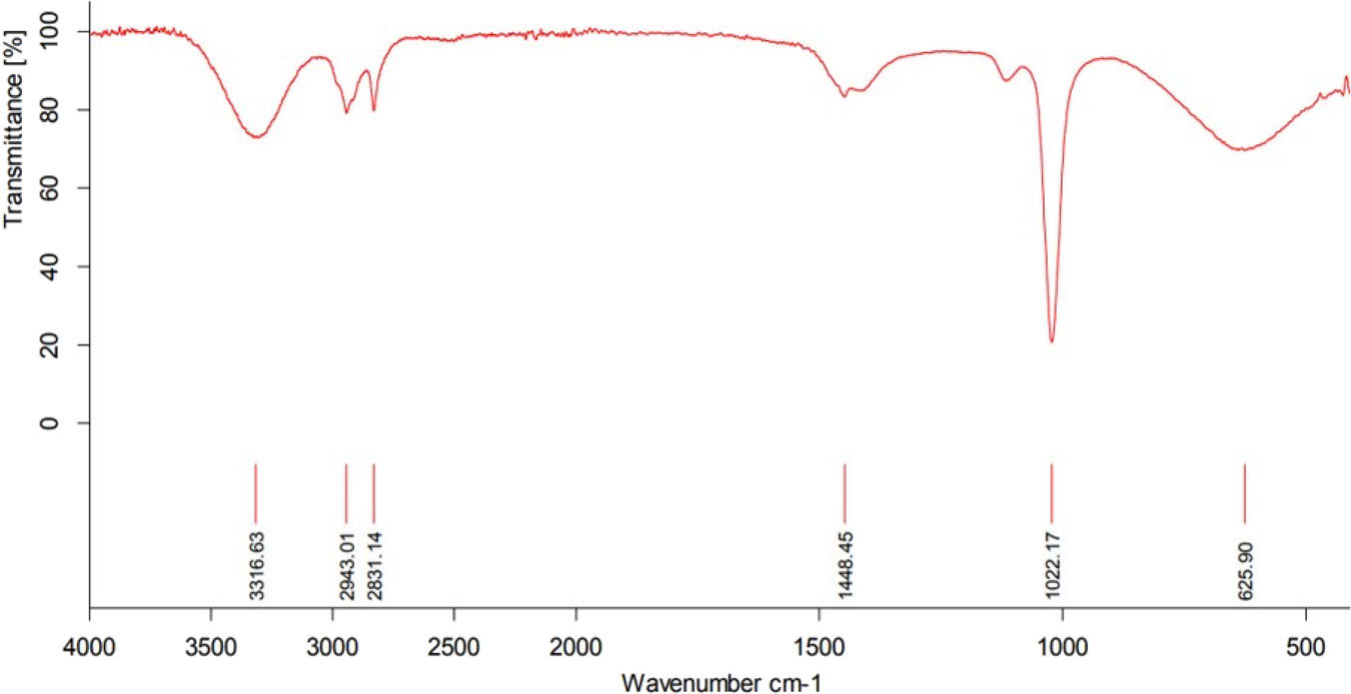
FT-IR of CuO/GO nanocomposites

It could be seen that the vibrational modes at 606 and 508 cm-1 indicate the Cu-O vibrations in the FT-IR plots, as shown in Fig. 1. The peaks in the range of 1300–1420 cm^−1^ and 2919 cm^−1^ indicate the stretching vibrations of C-H bending. C-O stretching at 1046 cm-1 has also been noticed in the FT-IR plot. It was interesting to note that no Cu_2_O stretching modes were detected in the plot. The broad stretching vibrational modes at 3411 and 1631 cm^−1^ indicate the O-H vibrations. Such results clearly stated the formation of hydration layers over the CuO surfaces during the nanocomposite synthesis.

### Nuclear magnetic resonance (NMR)

**Fig. 2:**
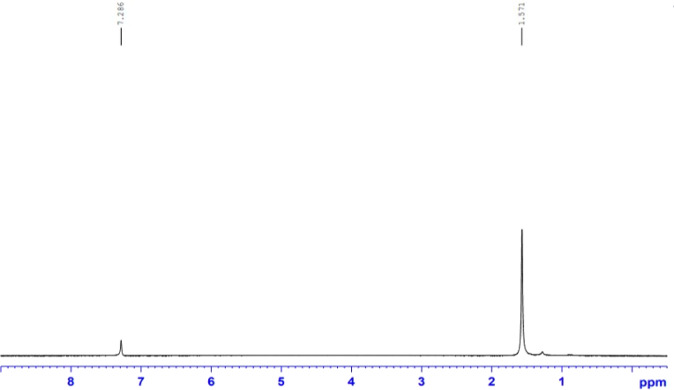
^13^CNMR spectra of CuO/GO nanocomposites

The as-prepared nanocomposites clearly show the formation of differential peak heights in C^13^ NMR plot as shown in Fig. 2 for different ppm for the formation of local magnetic moment around the nuclei of each participating atomic nuclei of the nanocomposites of Copper and Carbon, respectively. Such intermixing magnetic moment states in the ^13^C NMR plot exhibit the successful formation of the desired nanocomposites. Further exploration of the interacted oxidation states using X-ray Photoelectron Spectroscopy (XPS) of Cu and Carbon would help uncover the mixture’s real oxidation states.

### FE-SEM analysis

**Fig. 3:**
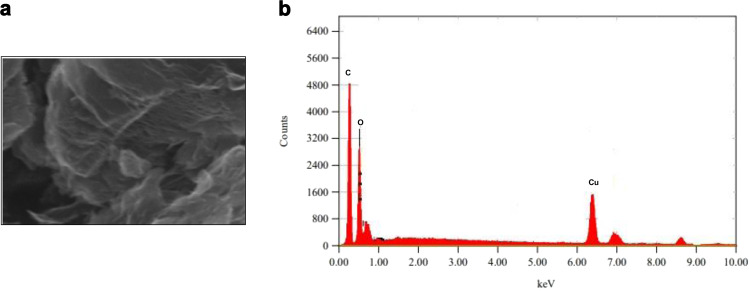
FE-SEM and EDAX spectra of CuO/GO nanocomposites

As shown in Fig. 3, the characteristic FE-SEM analyses could be noticed that successful formation of the CuO/GO sheets has been formed. The results are in parity with the previous publications in similar fields. The surface morphological features of the CuO /GO sheets have been substantiated using EDAX analyses, which exhibited Cu peaks and C peaks in good proportions.

### HR-TEM analysis

**Fig. 4:**
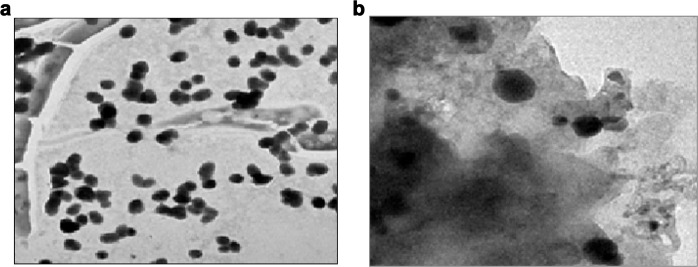
HR-TEM of CuO/GO nanocomposites indicating the CuO deposits over GO sheets (indicated by arrows)

The internal morphology of the as-prepared nanocomposites has exhibited an enwrapped GO sheets over the CuO nanoparticles, as seen in Fig. 4. The images could be easily seen for the formation of the nanocomposites in better proportions. The same features in the HRTEM images indicate that CuO nanoparticles have been doped and decorated over the GO sheets in an integrating manner.

### Antibacterial activity

**Fig. 5:**
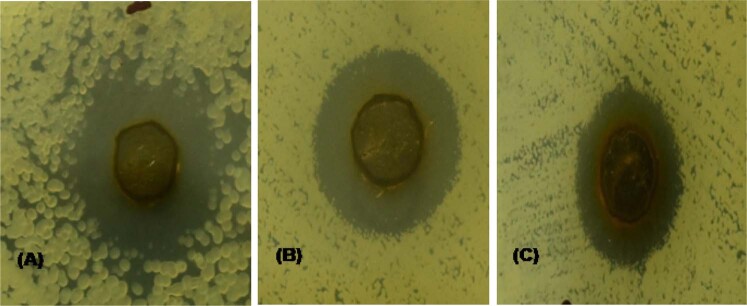
Antibacterial activity in agar well diffusion method (A) *E. coli* (B) *S. aureus* (C) *P. aeruginosa*

The antibacterial activity of CuO/GO nanocomposite was preliminarily determined by agar well diffusion (AWD) method; against three human pathogenic bacteria *S. aureus, E. coli* and *P. aeruginosa* as shown in Fig. 5 & Table 1. From the zone of inhibition, it was found that CuO/GO nanocomposite has the highest activity against *E. coli*. Further, the antibacterial assay was carried out by using the micro broth dilution method to corroborate the results found in the AWD method. Table 1 illustrates the effective percentage (%) inhibition and the respective MIC of each strain. It could be noticed from AWD method that *E. coli* among all the selected bacterial strains exhibited an augmented bactericidal inhibition %. In contrast, all three strains exhibited a more than 90 % inhibition rate. Among the three MIC studies as shown in Table 1, IC_50_ calculations show a pronounced antibacterial activity of *B. subtilis* (90.67 ± 0.32 µg/ml), *P. aeruginosa* (114.95 ± 0.79 µg/ml), and against *E. coli* (72.98 ± 0.27 µg/ml), which has been supported and corroborated with several previous publications in the similar lines.

**Table 1:** Antibacterial activity of CuO/GO nanocomposite

Name of the test strain	Mean zone of inhibition ± SD (in mm)	Percentage of inhibition (%) ± SD	MIC IC_50_ (µg/ml)
*Staphylococcus aureus*	18.57 ± 0.05	94.03 ± 0.54	90.67 ± 0.32
*Escherichia coli*	20.10 ± 0.16	99.37 ± 0.17	72.98 ± 0.27
*Pseudomonas aeruginosa*	15.57 ± 0.04	91.37 ± 0.70	114.95 ± 0.79

It could be understood from the observations of antibacterial assays that CuO nanoparticles exhibited a broad spectrum antibacterial potentiality against Gram-positive and Gram-negative strains [11].

The exact mechanism of nanomaterials-mediated potential antibacterial activity is not appropriately established. However, various possible mechanisms were put forwarded in the research reports regarding the antibacterial effect of nanomaterials. It was proposed that nanomaterial permeate the cell wall of the bacteria because of their adhering ability, eventually altering the bacterial membranes’ structural integrity and resulting in penetration inside the bacterial cells leading to cidal activity of the bacterial cells [23].On the other hand, it has also been speculated by several studies that the permeated nanomaterials inside the bacterial cells cause the net interactions with the vital macromolecules like DNA, proteins of the bacterial cells resulting in the production of Reactive Oxygen Species radicals causing degradation of the cellular integrity leading to death of the [24]. Several cysteine bonds and other enzymatic importance bonds are vulnerable to attack by several nanomaterials, leading to the deformations in the bacterial cells’ intrinsic bonding patterns, resulting in breakage and denaturation causing a death response the bacterial cells against the nanomaterials [25]. It has also been reported that several nanomaterials of importance attach the intermittent sulphur and phosphorous groups in the DNA molecules of the bacterial cells, resulting in deformations in the basic architecture of the bacterial cells leading to death [26]. Such augmented antibacterial activities achieved for the CuO/GO nanocomposites could be harnessed for designing an effective therapeutic and harbouring diagnostic attributes device, which can aid in the fight against drug-resistant medically important microbes [27].

### Underlying mechanism of antibacterial activity and FESEM analyses

**Fig. 6:**
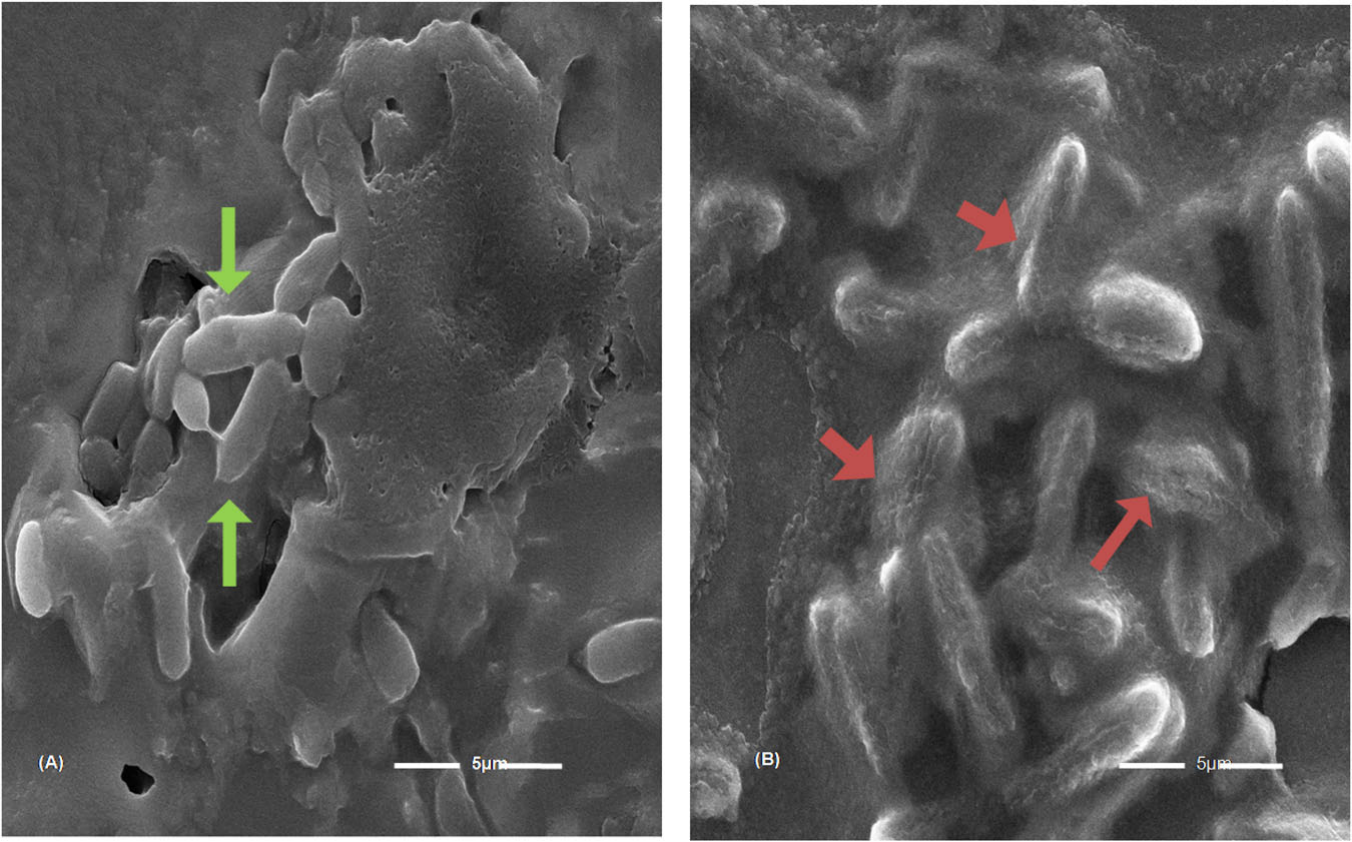
FE-SEM micrographs of untreated *E. coli* cells (**A**) showing intact and high electron density morphology (green arrow). (**B**) The considerable size of adhered nanocomposites was observed (brick red arrow) attached to the surface of the cells of the bacteria, and disrupted cell wall and membrane leakage was observed. The scale bar is 5 μm

In order to understand the nature of the interaction between the bacterial cells and the as-prepared nanocomposites, the ultrastructural aspects of the treated bacterial cultures and the respective control cultures were determined using FE-SEM analyses as seen in Fig. 6. It could be clearly understood from Fig. 6 that upon getting into contact with the nanocomposites, the uniform nature of the surface of *E. coli* has been seemed to be corrugated as when compared to the untreated cultures of the *E. coli* culture. Such induction in the unevenness in the bacterial outer surface architecture is mainly because of the fact that upon CuO/GO nanocomposite interactions at the outer membrane of the bacterial surface, there is an interplay of redox reaction between the composite and the bacterial cellular outer membranes. The resulting oxidation/ reduction process causes a net rupturing in the uniform cellular architecture of the outer bacterial membrane, causing a state of vulnerability in the bacterial membranes for permeating the nanocomposites inside the bacterial interior, resulting in destabilizing the inner chemistry of the cells causing some augmented bactericidal responses.

### Antioxidant activity

The antioxidant activity assessment of nanomaterials or nanocomposites is one of the most crucial studies prior to use in any biomedical application system [28]. Antioxidants have a significant impact on the performance of all bio-systems [29]. The generation of free radicals is very frequent in biological systems due to the interaction of biomolecules present in the system with molecular oxygen [30]. The antioxidant activity of synthetic nanomaterials has been evaluated before its real-time application concerning any living system (Brand-Williams et al., 1995). The DPPH scavenging assay is prevalent in evaluating the antioxidant property of newly formed materials utilizing tests that employ DPPH radicals. In the DPPH assay, the antioxidant entities present in the test sample reduce the stable nitrogen radical DPPH and produce the reduced form of DPPH by donating oxygen atoms. In the current work, the DPPH scavenging of CuO/GO nanocomposite was determined by estimatingIC_50_parameter, which illustrates the capability to scavenge free radicals.

**Fig. 7:**
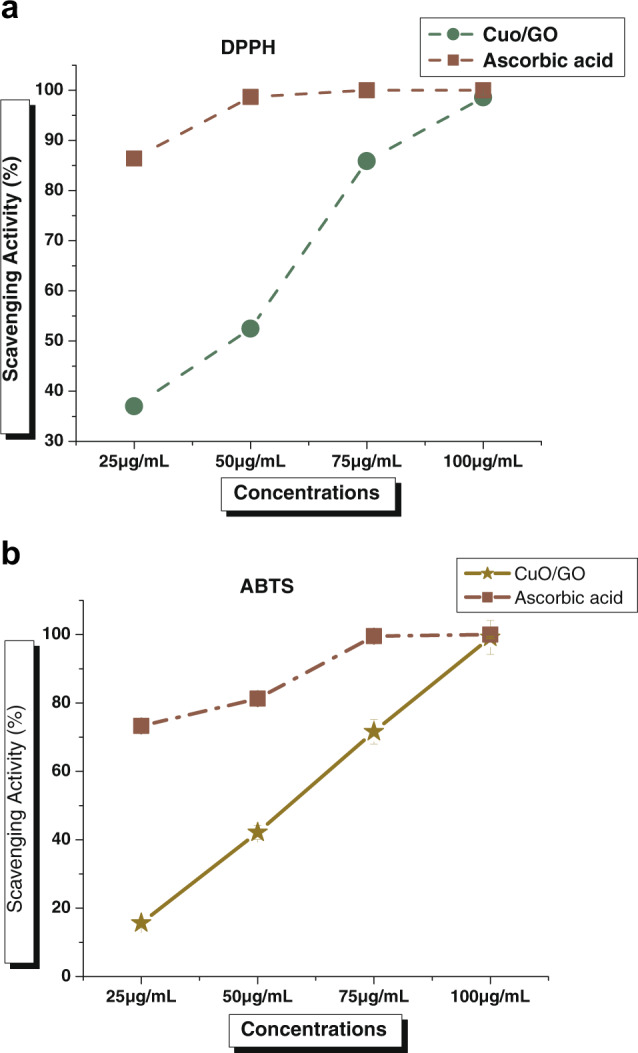
Antioxidant activity of CuO/GO nanocomposite in terms of radical scavenging activity (**A**) DPPH and (**B**) ABTS. Error bar represents the standard deviation of the mean. *p ≤ 0.05

For CuO/GO nanocomposite, theIC_50_value was 44.86 ± 1.74 μg/ml (Fig. 7), indicating significant antioxidant potential in DPPH scavenging. Additionally, the ABTS radical scavenging activity was also studied to prove more safe and potential use of CuO/GO nanocomposite in biomedical devices for efficient applications in different bio applications. The IC_50_value was calculated as 55.70 ± 0.19 μg/ml (Fig. 7) for CuO/GO nanocomposite in ABTS radical scavenging activity. Previously, there were many reports have been published regarding the potential antioxidant activities by using individual CuO nanoparticles and GO nanosheets, and the current antioxidant results were highly supported by the previous studies [18, 31].

### Cytotoxic activity against A-431 cell lines

**Fig. 8:**
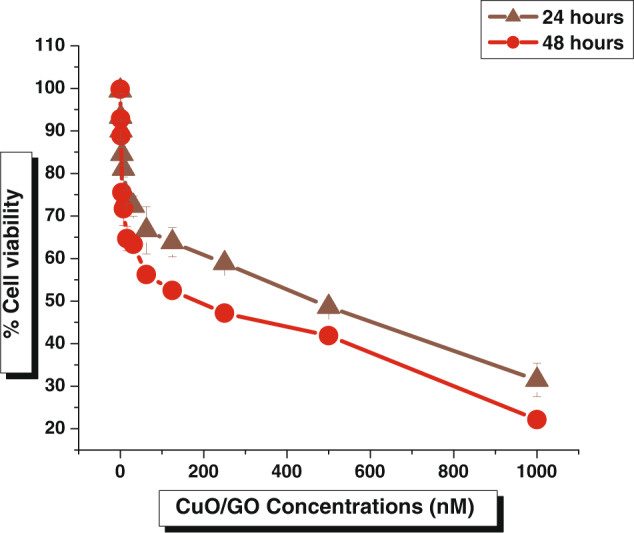
Cell viability of A-431 after treatment with different CuO/GO nanocomposites concentrations after 24 and 48 h. Error bar represents the standard deviation of the mean *p ≤ 0.05

The A-431cancer cells were treated with CuO/GO nanocomposites in the culture at varying concentrations (0.97 nM to 1000 nM) for 24 h at 37 °C. The methylthiazolyldiphenyl-tetrazolium bromide [MTT] assay was used to evaluate epidermoid carcinoma (A-431) cell viability upon treatment with CuO/GO nanocomposites (Fig. 8). The concentration-dependent effect of CuO/GO nanocomposites on A-431 cell viability was observed from assay results. The percentage of cell viability, however, slowly reduced as the CuO/GO concentration increased. The treatment of the cancer cells with a ~1000 nM CuO/GO for 24 h decreased cell viability to ~31.46%.

**Fig. 9:**
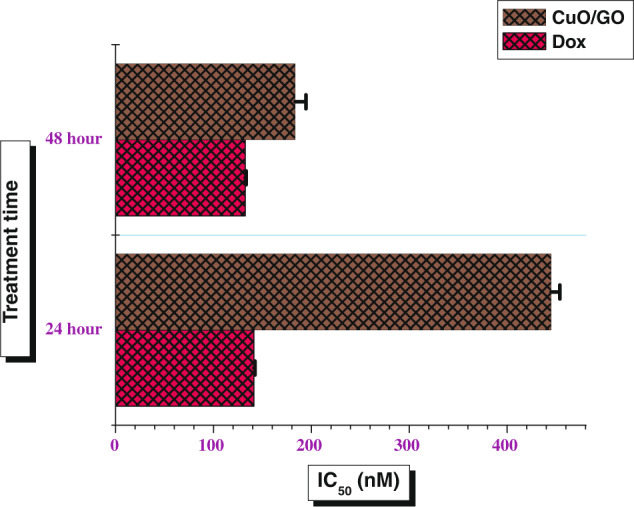
Cell viability assay of A-431 after treatment with CuO/GO nanocomposite and doxorubicin (control) in IC_50_ calculations. Error bar represents the standard deviation of the mean *p ≤ 0.05. Significant difference (*p* ≤ 0.05) within a parameter between two lines is denoted by an asterisk

It could be observed that upon an increase in the concentration of the CuO/GO nanocomposites, the percentage of the viable cancer cells seems to be concomitantly reduced. It is observed that the percentage of viable cells of ~48.63% and 81.06%, respectively were detected when the cells were treated with 500 nM and 7.8 nM concentrations of CuO/GO nanocomposites after 24 h incubation. On the other hand, as shown in Fig. 9, upon treatment of the test solution cells with the nanocomposites of same concentration ~ 500 nM and 7.8 nM, the viability seems to be reduced slightly of ~41.86 and 71.67% with the incubation timings after 48 h. Such differential cytotoxic responses indicate that a dose-dependent response of A-431 cancer cells exhibits a negligible influence of time-dependent cytotoxicity responses when tested in similar cell lines at the same concentration dosages in-vitro.

Many previous research reports have supported time-dependent and dose-dependent cytotoxicity against A-431cells [31, 32]. It has been investigated that ROS formation during the cellular metabolism takes place after expose to test sample, plays a vital role in the mechanism of cytotoxicity of CuO/GO nanocomposites in epidermoid carcinoma [A-431] [33]. It has been further speculated that upon introduction of the CuO/GO nanocomposites into the cells, the intrinsic vital organelle of the cells, also known as the ‘*power house*’ of the cell ‘*mitochondrion*’ is also gets targeted severely because of the formation of free radicals of O^-^,O^2-^ etc. causing a disability in the essential metabolism in the cells, leading to the death of the cells [34].

The IC_50_ value of CuO/GO was found to be 444.96 ± 8.87 nM in 24 h and 183.51 ± 10.99 nMin 48 h of treatment in A-431 cells. However, theIC_50_ of doxorubicin [control] was found 141.33 ± 1.00 nM in 24 h and 132.46 ± 0.94 nMin 48 h of exposure to the cancer cells. It was observed that the IC_50_value of CuO/GO is almost very similar to doxorubicin in 48 h of exposure (Fig. 9). Hence, CuO/GO nanocomposites can be considered potential candidates for the oncology treatments application [31].

## Conclusion

The as-prepared nanocomposites exhibited tremendous antimicrobial and antioxidant implications when tested in-vitro, forming a part of the therapeutic activity of the nanocomposites. The average particle size of the formed nanocomposites came about ~20 nm, which is significant enough to elicit biological activities when tested. The possible diagnostic and drug-target interaction for designing a possible theranostic agent has been tried to be explained using mathematical modelling of drug-target interactions and its underlying pharmacodynamics phenomenon. The pristine electrochemical, Physico-chemical properties of CuO nanoparticles, along with the water-soluble GO, makes the composite a robust platform for designing futuristic nano-electromechanical devices which can be used for sensing the slightest analytes by tuning the pristine direct band gap features and p-type nature of the CuO nanoparticle entity in the biomedical domain.
